# Aplasia Cutis Congenita Type V Associated With Fetus Papyraceus in a Dichorionic Diamniotic Twin Pregnancy

**DOI:** 10.1155/crpe/9504629

**Published:** 2025-04-28

**Authors:** Julie Baverman, Mariah Fleischman, David Brooks

**Affiliations:** Department of Pediatrics, Creighton University School of Medicine, Omaha, Nebraska, USA

**Keywords:** aplasia cutis congenita type V, fetus papyraceus, stellate lesions, vanishing twin

## Abstract

A male neonate was born at 40 weeks and 3 days gestation with bilateral, stellate shaped truncal lesions, consistent with type V aplasia cutis congenita (ACC). The infant was the survivor of a dichorionic diamniotic twin pregnancy, with fetal demise documented at 13 weeks gestation. Here we present a unique case of ACC associated with fetal papyraceus, along with a review of the current literature on this heterogeneous group of disorders.

## 1. Introduction

Aplasia cutis congenita (ACC) is the absence of skin at birth, presenting anywhere on the cutaneous surface of the body [1]. The term ACC encompasses a large group of disorders that was first classified into nine subsets in 1986 by Frieden (Table 1) [2]. The dominant majority (85%) of cases present as scalp lesions [3]. While the exact pathophysiology resulting in ACC remains unclear, the absence of skin is speculated to result from an in utero developmental disruption [2]. With type V ACC, the disruption is suspected to be due to ischemic or thrombotic events in utero following the death of a twin [2–4]. The trunk is often involved in type V ACC as the blood vessels supplying this region are vulnerable to hypoperfusion during periods of placental ischemia [5]. Type V ACC is associated with fetus papyraceus, a term describing the early intrauterine demise of a twin, i.e., “a vanishing twin,” resulting in the absorption and mechanical compression of the deceased fetus into the remaining fetus [6, 7]. ACC type V is rare, affecting approximately 0.5–3 in 10,000 live births, with 90%–95% of these cases resulting from monochorionic pregnancies [3, 8]. Here we describe a unique case of type V ACC in the surviving twin of a dichorionic diamniotic twin pregnancy.

## 2. Case Presentation

A male infant at 40 weeks and 3 days gestation was born to a teen, primigravida mother via spontaneous vaginal delivery after a scheduled induction of labor. The newborn was a result of a spontaneous dichorionic diamniotic intrauterine pregnancy. Prenatal care was initiated at 13 weeks 3 days with an ultrasound demonstrating Twin B with an absent heartbeat (fetal demise) and Twin A with echogenic intracardiac foci (EIF). There was a small growth discordance between the twins suggesting the demise of Twin B occurred recently. The pregnancy was followed by maternal fetal medicine (MFM). Due to the echogenic focus, genetic testing was offered to the mother but was declined. Twin A had adequate growth throughout the pregnancy, with a birth weight of 3710 g. There were no additional anomalies noted on serial MFM ultrasounds and no further mention of the demised fetal tissue.

The maternal history was significant for teen pregnancy and recurrent UTIs. During pregnancy, the mother took prophylactic aspirin for pre-eclampsia prevention, vitamin B12, and a prenatal vitamin. Prenatal labs were unremarkable including varicella immune from prior immunization.

**Figure 1:**
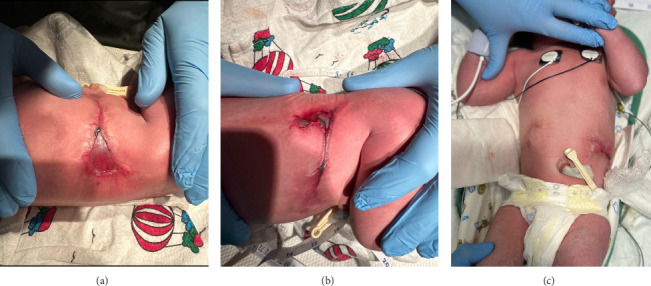
Cicatrix scarring along bilateral flanks with small triangular regions of translucent skin covering the lesions. (a) Right flank scar. (b) Left flank scar. (c) Anterior view of bilateral scars.

Delivery of the male infant was complicated by chorioamnionitis and shoulder dystocia. NICU was called to the delivery due to shoulder dystocia. The infant emerged vigorous with a strong cry and was brought to the radiant warmer where he was noted to have cicatrix scarring along his flanks bilaterally, both with small triangular regions of translucent skin (Figure 1). No identified tissue of the demised fetus was present on the surviving infant or attached to the placenta.

The infant was admitted to the NICU. Physical exam outside of the dermatologic findings was normal without any physical features consistent with trisomy 21. Due to chorioamnionitis and the translucent skin lesions, a blood culture was obtained and the infant received 36 h of antibiotics. Plastic surgery was consulted and recommended conservative management with bacitracin and Xeroform dressings. While in the NICU, the infant ate well with normal voiding and stooling. An echocardiogram was obtained due to the prenatal concern for intracardiac echogenic foci and it was not visualized on the postnatal echocardiogram. The infant was discharged home on day of life 3. The patient was seen in follow-up in plastic surgery clinic with complete resolution of the lesions.

## 3. Discussion

ACC is a rare and heterogeneous group of congenital disorders, first documented by Cordon in 1767 [8]. ACC is characterized by the localized or widespread absence of skin layers which can include the epidermis, dermis, subcutaneous tissue, and, in rare cases, bone. While the mechanism of injury is uncertain, several etiologies have been proposed including chromosomal abnormalities, ischemic or thrombotic events, teratogenic drug exposure, intrauterine infection, and trauma [9].

The classification system developed by Frieden in 1986 categorizes ACC based on lesion location and associated congenital anomalies. Categorizing this large group of disorders into nine distinct subsets enhances understanding of the wide variety of clinical presentations of ACC, thus allowing for more accurate diagnosis and preventing treatment delay [10]. ACC is a clinical diagnosis and often is not noted on prenatal ultrasound unless lesions are extensive [3, 9, 11]. Because prenatal detection is limited, it is important for clinicians to accurately recognize the various presentations of ACC during the immediate neonatal period.

Type V ACC encompasses cases with truncal lesions that are closely linked to placental infarcts and vanishing twins. The vascular theory offers a plausible explanation for these lesions, proposing that rapid exsanguination creates a shunting effect, directing blood from the surviving twin to the relaxed circulation of the dying twin [5, 7, 12]. The resulting hypovolemic state of the surviving twin causes under-perfusion in critical watershed areas of the skin, leading to ischemic changes, necrosis, and ultimately, the characteristic truncal ACC lesions [3, 13]. As in our case, type V ACC exhibits a distinctive, reproducible pattern of bilateral, symmetric lesions typically located on the trunk or thighs [7]. The symmetrical presentation aids in distinguishing type V ACC from other forms and underscores the likely role of specific intrauterine events—such as placental anomalies or a resorbed twin—in its pathogenesis [2].

Type V ACC is a rare phenomenon with approximately 100 cases reported in the literature [10], and approximately 95% of these cases are associated with fetal demise in the first trimester of a monochorionic pregnancy [3]. Dichorionic pregnancies are usually protected against hypoperfusion watershed infarcts that occur with fetal demise as each twin has its own placental blood supply resulting in less vascular interdependence [12]. However, as in our case, type V ACC has been observed in surviving twins of dichorionic pregnancies [14].

Treatment for ACC type V is typically conservative, with proper wound care to prevent infection or fluid loss [10]. In more extensive presentations, plastic surgery or grafting may be warranted to repair the cutaneous defects. Regardless, prompt assessment and diagnosis are important so that appropriate management strategies are initiated.

EIF are a nonspecific finding on prenatal ultrasound and are considered a “soft” marker for trisomy 21. They are observed in 15%–30% of fetuses with trisomy 21 and 4%–7% of euploid fetuses. As an isolated finding, aneuploidy testing should be offered. In a euploid fetus or infant without additional features consistent with trisomy 21, EIF should be considered a normal variant [15]. EIF often resolve on postnatal echocardiogram and are not associated with major intracardiac or extracardiac abnormalities [16].

## 4. Conclusions

ACC is an overarching term that encompasses a wide variety of clinical presentations and genetic associations. Type V ACC is a clinical diagnosis and is predominantly managed conservatively, with surgical intervention reserved for cases with extensive cutaneous involvement. Here we report a rare case of type V ACC presenting uniquely in a dichorionic twin gestation. This case contributes to the growing body of reports demonstrating the various presentations of ACC.

## Figures and Tables

**Table 1:** 1986 classification of ACC by Frieden.

I—ACC on scalp with no associated anomalies
II—ACC on scalp with associated limb anomalies
III—ACC on scalp with associated epidermal and organoid nevi and possible seizures
IV—ACC of any site with associated embryological anomalies
V—ACC of any site with associated fetal papyraceus or placental infarcts
VI—ACC of extremities with associated epidermolysis bullosa
VII—ACC of extremities with no associated blistering
VIII—ACC of any site caused by specific teratogens
IX—ACC of any site with associated malformation syndromes

## Data Availability

Data sharing is not applicable to this article as no datasets were generated or analyzed during the current study.
